# Multi-Sensor Optimal Data Fusion Based on the Adaptive Fading Unscented Kalman Filter

**DOI:** 10.3390/s18020488

**Published:** 2018-02-06

**Authors:** Bingbing Gao, Gaoge Hu, Shesheng Gao, Yongmin Zhong, Chengfan Gu

**Affiliations:** 1 School of Automatics, Northwestern Polytechnical University, Xi’an 710072, China; hugaoge1111@126.com (G.H.); gshshnpu@163.com (S.G.); 2 School of Engineering, RMIT University, Bundoora, VIC 3083, Australia; yongmin.zhong@rmit.edu.au (Y.Z.); chengfan.gu@gmail.com (C.G.)

**Keywords:** multi-sensor data fusion, adaptive fading unscented Kalman filter, process-modeling error, Mahalanobis distance, linear minimum variance

## Abstract

This paper presents a new optimal data fusion methodology based on the adaptive fading unscented Kalman filter for multi-sensor nonlinear stochastic systems. This methodology has a two-level fusion structure: at the bottom level, an adaptive fading unscented Kalman filter based on the Mahalanobis distance is developed and serves as local filters to improve the adaptability and robustness of local state estimations against process-modeling error; at the top level, an unscented transformation-based multi-sensor optimal data fusion for the case of *N* local filters is established according to the principle of linear minimum variance to calculate globally optimal state estimation by fusion of local estimations. The proposed methodology effectively refrains from the influence of process-modeling error on the fusion solution, leading to improved adaptability and robustness of data fusion for multi-sensor nonlinear stochastic systems. It also achieves globally optimal fusion results based on the principle of linear minimum variance. Simulation and experimental results demonstrate the efficacy of the proposed methodology for INS/GNSS/CNS (inertial navigation system/global navigation satellite system/celestial navigation system) integrated navigation.

## 1. Introduction

With the rapid development of electronics technologies, various sensors have been developed and applied to many engineering fields such as integrated navigation, target tracking, signal processing, and networked communications [1,2,3,4]. Since the use of multiple sensors provides more accurate and reliable information than that of a single sensor alone, multi-sensor data fusion has received considerable attention in recent years. The basic idea of multi-sensor data fusion is to combine the data obtained from multiple sensors and further associate them with database information to achieve improved estimation accuracy compared to the use of a single sensor [5,6].

The existing studies on multi-sensor data fusion can be divided into two categories [4,6]. One is the centralized fusion method, which can achieve a globally optimal state estimation by directly combining local measurement models to form an augmented measurement model [7,8]. This method has minimal information loss. However, it causes large computation and communication burdens in the fusion center due to high-dimension computations and large data memories. Further, it has a poor robustness and reliability when one sensor is faulty [1,9]. The other is the decentralized fusion method, which can yield globally optimal or suboptimal state estimations according to certain criteria [9,10]. This method has small computation and communication burdens in the fusion center due to the decentralized structure. Furthermore, the decentralized structure also enables easy fault detection and isolation. Due to its superiority over the centralized fusion method, the decentralized fusion method has been extensively used in engineering fields.

The federated Kalman filter (FKF) is a typical example of the decentralized fusion method. It employs the principle of information sharing for local and global filters, and eliminates the correlation between local estimations by using the technique of covariance upper bound [11,12]. However, FKF is only suitable for multi-sensor linear stochastic systems [13,14]. Its fusion accuracy is sharply deteriorated when applied to multi-sensor nonlinear stochastic systems. In addition, due to the use of process noise covariance’s upper bound rather than process noise covariance itself, the resultant local state estimations are at suboptimal level, making the global state estimation also at suboptimal level. Furthermore, FKF also requires the local estimations resulting from local filters to be independent initially, which is difficult to implement in practical engineering [4,14,15].

The unscented Kalman filter (UKF) is a promising filtering method to estimate the state of a nonlinear stochastic system [16,17]. This method can approximate the posterior mean and covariance of any Gaussian random variable in third-order accuracy by using unscented transformation (UT). It has advantages of high estimation accuracy, high convergence rate and simple implementation compared to other nonlinear filtering methods [17,18]. Due to these merits, Hu and Huang [13] presented an UKF-based FKF (UKF-FKF) by combining UKF with FKF to address the data fusion problem in multi-sensor nonlinear stochastic systems. This method adopts UKFs as local filters to calculate local state estimations, avoiding the linearization error of the system model involved in FKF and improving the estimation accuracy of local filters. However, it still suffers from the problem due to the use of covariance upper bound in FKF. Further, since UKF is designed based on the condition that the nonlinear system can be exactly modeled [19,20,21], UKF-FKF also requires the system model to be accurate. If the system model of a multi-sensor nonlinear stochastic system involves modeling error, the performances of local filters in UKF-FKF will be degraded or even divergent, leading to the poor fusion accuracy.

Currently, the existing multi-sensor data fusion methods are mainly dominated by inhibiting the influence of covariance upper bound on the fusion accuracy. Chang et al. [22] studied an information matrix-based distributed fusion Kalman filter. The authors’ group also reported a random weighting estimation-based multi-sensor optimal data fusion approach and a matrix weighted multi-sensor data fusion method [23,24]. However, the above studies are mainly focused on multi-sensor linear stochastic systems. Just recently, the authors’ group reported a UKF-based multi-sensor optimal data fusion method (UKF-MODF) for multi-sensor nonlinear stochastic systems to inhibit the influence of covariance upper bound on the fusion solution [14]. This method develops an optimal data fusion scheme according to the principle of linear minimum variance to overcome the problem due to the use of covariance upper bound in FKF. It can achieve the globally optimal state estimation for multi-sensor nonlinear stochastic systems. However, since UKF still serves as local filters to calculate local state estimations, the estimation accuracy of UKF-MODF also relies on the accuracy of the system model. Furthermore, the global state estimation process of UKF-MODF is only suitable for the case with two local filters. It is necessary to provide a process of global state estimation for general cases with *N* local filters.

The system model of a nonlinear stochastic system consists of process and measurement models. In practical applications, the measurement model’s accuracy can be guaranteed by using high-precision measurement equipment together with plenty of available measurement data [25,26]. However, since the process model is a theoretical approximation to the real-world dynamic system [26], it inevitably involves error. In some applications with the preference of computational simplicity and real-time performance, such an approximation is intended given the complexity of the dynamic environment [25]. Therefore, the process-modeling error is generally considered to be the main source of model error for a nonlinear stochastic system [25,26,27,28]. 

Adaptive UKF is a strategy to improve the UKF adaptability and robustness against process-modeling error by adjusting the Kalman gain matrix for state estimation. Song and Han presented an adaptive UKF to inhibit the process-modeling error by updating the process noise covariance via minimization of the difference between computed and actual innovation covariances [29]. However, this method requires the calculation of partial derivatives, causing a relatively large computation burden. Meng et al. reported a covariance matching-based adaptive UKF to make online estimates of and adjust process noise covariance using innovation and residual sequences [30]. However, this covariance matching technique yields a steady-state estimation error, leading to a limited improvement in the filtering accuracy [20]. Compared to the above two adaptive UKFs, the adaptive fading UKF (AFUKF) can effectively inhibit the influence of process-modeling error on the filtering solution under a modest computation load [27,28]. In this method, when process-modeling error is detected, an adaptive fading factor is constructed based on innovation vector and its corresponding statistical information. By using the adaptive fading factor to scale process noise covariance or predicted state covariance, historical information is less weighted while current measurement information is more weighted to tune UKF recursively to alleviate the influence of process-modeling error on the estimation solution [19,28]. Therefore, the introduction of AFUKF into multi-sensor optimal data fusion provides a solution to improve the adaptability and robustness of UKF-MODF.

This paper focuses on improvement of our previous work (UKF-MODF) for multi-sensor nonlinear stochastic systems. It presents a novel multi-sensor optimal data fusion methodology based on AFUKF to address the UKF-MODF problems, leading to improved adaptability and robustness of data fusion for multi-sensor nonlinear stochastic systems. This methodology is in a two-level fusion structure: at the bottom level, an AFUKF based on the Mahalanobis distance is developed and serves as local filters to improve the adaptability and robustness of local state estimations against process-modeling error; at the top level, an UT-based multi-sensor optimal data fusion for the case of *N* local filters is established according to the principle of linear minimum variance to calculate globally optimal state estimation via fusion of local estimations. The proposed methodology not only improves the robustness of multi-sensor data fusion against process-modeling error, but it also achieves globally optimal fusion results based on the principle of linear minimum variance. Simulations and practical experiments as well as comparison analysis with FKF, UKF-FKF and UKF-MODF have been conducted to comprehensively evaluate the performance of the proposed multi-sensor optimal fusion methodology for INS/GNSS/CNS (inertial navigation system/global navigation satellite system/celestial navigation system) integrated navigation system.

## 2. Adaptive Fading UKF Based Multi-Sensor Optimal Data Fusion 

**Figure 1:**
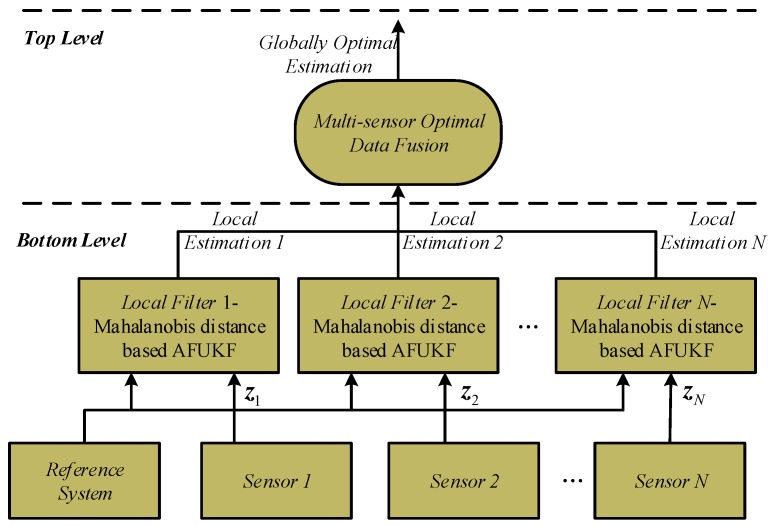
The framework of the proposed AFUKF-based multi-sensor optimal data fusion.

The framework of the proposed AFUKF-based multi-sensor optimal data fusion methodology (AFUKF-MODF) is shown in Figure 1. It has a two-level fusion structure: at the bottom level, an AFUKF based on the Mahalanobis distance is developed and serves as local filters to improve the robustness of local state estimations against process-modeling error; at the top level, according to the principle of linear minimum variance, the UT-based multi-sensor optimal data fusion for the case of *N* local filters is established to calculate the globally optimal state estimation by fusion of local estimations.

### 2.1. Local State Estimation

Consider the ith (i=1,2,⋯,N) local filter with the following dynamic model

(1) $$\{\begin{array}{c} x(k)=f\left(x(k-1)\right)+w(k)\\ z_{i}(k)=h_{i}\left(x(k)\right)+v_{i}(k) \end{array}$$

where $x(k)\in R^{n}$ is the system state vector; f(⋅) is the nonlinear state function; w(k) is the process noise which is commonly assumed as a zero-mean Gaussian white noise with covariance Q≥0; $z_{i}(k)\in R^{m_{i}}$ is the measurement of the ith local filter; $h_{i}(\cdot )$ is the nonlinear measurement function of the ith local filter; and $v_{i}(k)$ is the measurement noise of the ith local filter and is commonly assumed as a zero-mean Gaussian white noise with covariance $R_{i}>0$. 

#### 2.1.1. Classical UKF

In order to describe the Mahalanobis distance-based AFUKF clearly, let us briefly review the classical UKF at first. For the nonlinear system model given by (1), the procedure of the classical UKF can be summarized as:

**Step 1:** *Initialization*

(2) $$\{\begin{array}{c} {\hat{x}}_{i}(0)=E[x_{i}(0)]\\ P_{i}(0)=E\left[\left(x_{i}(0)-{\hat{x}}_{i}(0)\right){\left(x_{i}(0)-{\hat{x}}_{i}(0)\right)}^{T}\right] \end{array}$$

**Step 2:** *Time Update*

Assume that the state estimate ${\hat{x}}_{i}(k-1)$ and the corresponding error covariance matrix $P_{i}(k-1)$ are given, the sigma points are selected as

(3) $$\left\{ \begin{array}{ll} \chi_{j,k-1}^{\left(i\right)}={\hat{x}}_{i}(k-1) & j=0\\ \chi_{j,k-1}^{\left(i\right)}={\hat{x}}_{i}(k-1)+a{\left(\sqrt{nP_{i}(k-1)}\right)}_{j} & j=1,2,\ldots ,n\\ \chi_{j,k-1}^{\left(i\right)}={\hat{x}}_{i}(k-1)-a{\left(\sqrt{nP_{i}(k-1)}\right)}_{j-n} & j=n+1,n+2,\ldots ,2n \end{array}\right.$$

where a is a tuning parameter to determine the spread of the sigma points around ${\hat{x}}_{i}(k-1)$ and it is commonly set to a small positive value; and ${\left(\sqrt{nP_{i}(k-1)}\right)}_{j}$ denotes the *j*th column of the square root of the matrix $nP_{i}(k-1)$.

Calculate the predicted state mean and covariance as

(4) $$\chi_{j,k/k-1}^{\left(i\right)}=f(\chi_{j,k-1}^{\left(i\right)})\;(j=0,1,\ldots ,2n)$$

(5) $${\hat{x}}_{i}(k/k-1)=\sum_{j=0}^{2n}\omega_{j}\chi_{j,k/k-1}^{\left(i\right)}$$

(6) $$P_{i}(k/k-1)=\sum_{j=0}^{2n}\omega_{j}(\chi_{j,k/k-1}^{\left(i\right)}-{\hat{x}}_{i}(k/k-1)){\left(\chi_{j,k/k-1}^{\left(i\right)}-{\hat{x}}_{i}(k/k-1)\right)}^{T}+Q$$

where $\left\{ \begin{array}{ll} \omega_{j}=1-\frac{1}{a^{2}}, & j=0\\ \omega_{j}=\frac{1}{2na^{2}}, & j=1,2,\cdots ,2n \end{array}\right.$.

**Step 3:** *Sigma Point Update*

Select a set of new sigma points with the mean ${\hat{x}}_{i}(k/k-1)$ and the covariance $P_{i}(k/k-1)$

(7) $$\left\{ \begin{array}{ll} {\chi^{\prime }}_{j,k/k-1}^{\left(i\right)}={\hat{x}}_{i}(k/k-1), & j=0\\ {\chi^{\prime }}_{j,k/k-1}^{\left(i\right)}={\hat{x}}_{i}(k/k-1)+{\left(a\sqrt{nP_{i}(k/k-1)}\right)}_{j}, & j=1,2,\ldots ,n\\ {\chi^{\prime }}_{j,k/k-1}^{\left(i\right)}={\hat{x}}_{i}(k/k-1)-{\left(a\sqrt{nP_{i}(k/k-1)}\right)}_{j-n}, & j=n+1,n+2,\ldots ,2n \end{array}\right.$$

**Step 4:** *Measurement Update*

The weighted mean and covariance of the predicted measurement are computed by

(8) $$\gamma_{j,k/k-1}^{\left(i\right)}=h({\chi^{\prime }}_{j,k/k-1}^{\left(i\right)})$$

(9) $${\hat{z}}_{i}(k/k-1)=\sum_{j=0}^{2n}\omega_{j}\gamma_{j,k/k-1}^{\left(i\right)}=\sum_{j=0}^{2n}\omega_{j}h({\chi^{\prime }}_{j,k/k-1}^{\left(i\right)})$$

(10) $$P_{i}({\hat{z}}_{i}(k/k-1))=\sum_{j=0}^{2n}\omega_{j}\left(\gamma_{j,k/k-1}^{\left(i\right)}-{\hat{z}}_{i}(k/k-1)\right){\left(\gamma_{j,k/k-1}^{\left(i\right)}-{\hat{z}}_{i}(k/k-1)\right)}^{T}+R_{i}$$

Subsequently, the state estimate and associated error covariance matrix are updated as

(11) $$P_{i}({\hat{x}}_{i}(k/k-1){\hat{z}}_{i}(k/k-1))=\sum_{j=0}^{2n}\omega_{j}\left(\chi_{j,k/k-1}^{\left(i\right)}-{\hat{x}}_{i}(k/k-1)\right){\left(\gamma_{j,k/k-1}^{\left(i\right)}-{\hat{z}}_{i}(k/k-1)\right)}^{T}$$

(12) $$K_{i}(k)=P_{i}({\hat{x}}_{i}(k/k-1){\hat{z}}_{i}(k/k-1))P_{i}^{-1}({\hat{z}}_{i}(k/k-1))$$

(13) $${\hat{x}}_{i}(k)={\hat{x}}_{i}(k/k-1)+K_{i}(k)(z_{i}(k)-{\hat{z}}_{i}(k/k-1))$$

(14) $$P_{i}(k)=P_{i}(k/k-1)-K_{i}(k)P_{i}({\hat{z}}_{i}(k/k-1))K_{i}^{T}(k)$$

**Step 5:** *Get back to Step 2 for the next sample until all samples are processed.*

#### 2.1.2. Mahalanobis Distance Based Adaptive Fading UKF

The Mahalanobis distance of innovation vector, which is based on hypothesis testing theory, is commonly used as a measure to identify system modeling error for Gaussian systems [27,31]. This paper adopts the Mahalanobis distance into AFUKF and further develop a Mahalanobis distance-based AFUKF to improve the adaptability and robustness of the classical UKF against process-modeling error for multi-sensor nonlinear stochastic systems. 

Define the innovation vector of the *i*th local filter as

(15) $${\tilde{z}}_{i}(k)=z_{i}(k)-{\hat{z}}_{i}(k/k-1)$$

For the nonlinear Gaussian system given by (1), ${\tilde{z}}_{i}(k)$ should obey the zero-mean Gaussian distribution with the covariance

(16) $$P_{i}({\hat{z}}_{i}(k/k-1))=\sum_{j=0}^{2n}\omega_{j}\left(\gamma_{j,k/k-1}^{\left(i\right)}-{\hat{z}}_{i}(k/k-1)\right){\left(\gamma_{j,k/k-1}^{\left(i\right)}-{\hat{z}}_{i}(k/k-1)\right)}^{T}+R_{i}$$

Thus, the square of the Mahalanobis Distance of the innovation vector should obey the chi-square distribution with $m_{i}$ degrees of freedom [27,31], i.e.,

(17) $$M_{i}^{2}(k)={\tilde{z}}_{i}^{T}(k){\left(P_{i}({\hat{z}}_{i}(k/k-1))\right)}^{-1}{\tilde{z}}_{i}(k)∼\chi_{m_{i}}^{2}$$

where $m_{i}$ is the dimension of the measurement in the ith local filter.

According to the hypothesis testing theory, for a given significance level α, we have

(18) $$P(M_{i}^{2}(k)\leq \chi_{m_{i},\alpha }^{2})=1-\alpha \;(0<\alpha \leq 1)$$

where P(⋅) represents the probability of a random event. 

If (18) holds, this means the system works under the optimal condition, i.e., the nonlinear multi-sensor system described by (1) does not have process-modeling error. However, if (18) does not hold, it can be concluded with high probability that there exists process-modeling error in the multi-sensor system (1). In this case, AFUKF incorporates a time-varying adaptive fading factor into the predicted state covariance matrix to refrain from the influence of prior knowledge of the current state estimate. Thus, the predicted state covariance matrix in AFUKF is modified as

(19) $$P_{i}^{*}(k/k-1)=\lambda_{k}^{i}\left(\sum_{j=0}^{2n}\omega_{j}(\chi_{j,k/k-1}^{\left(i\right)}-{\hat{x}}_{i}(k/k-1)){\left(\chi_{j,k/k-1}^{\left(i\right)}-{\hat{x}}_{i}(k/k-1)\right)}^{T}+Q\right)$$

Introduce the adaptive fading factor $\lambda_{k}^{i}$ to the predicted state covariance matrix such that (18) holds. Thus, we have the following equation

(20) $$g_{i}(\lambda_{k}^{i})={\tilde{z}}_{i}^{T}(k){\left(P_{i}^{*}({\hat{z}}_{i}(k/k-1))\right)}^{-1}{\tilde{z}}_{i}(k)-\chi_{m_{i},\alpha }^{2}=0$$

where $P_{i}^{*}({\hat{z}}_{i}(k/k-1))$ is the measurement prediction covariance matrix calculated by using $P_{i}^{*}(k/k-1)$.

It can be seen from (20) that the determination of the adaptive fading factor $\lambda_{k}^{i}$ is a problem of solving the nonlinear equation. Since the derivative of $g_{i}(\lambda_{k}^{i})$ with respect to $\lambda_{k}^{i}$ is difficult to calculate, the traditional Newton’s method cannot be used to solve the nonlinear Equation (20). In this paper, a chord secant method [32,33] is adopted to iteratively determine the adaptive fading factor $\lambda_{k}^{i}$ by solving the nonlinear Equation (20). Thus, we have

(21) $$\lambda_{k}^{i}(t+1)=\lambda_{k}^{i}(t)-\frac{g_{i}(\lambda_{k}^{i}(t))[\lambda_{k}^{i}(t)-\lambda_{k}^{i}(t-1)]}{g_{i}(\lambda_{k}^{i}(t))-g_{i}[\lambda_{k}^{i}(t-1)]},\;(t=1,2,\cdots )$$

where t represents the iteration time.

Substituting (20) into (21) yields

(22) $$\lambda_{k}^{i}(t+1)=\lambda_{k}^{i}(t)-\frac{\left[{\bar{M}}_{k}^{i}(\lambda_{k}^{i}(t))-\chi_{m_{i},\alpha }^{2}][\lambda_{k}^{i}(t)-\lambda_{k}^{i}(t-1)\right]}{{\bar{M}}_{k}^{i}(\lambda_{k}^{i}(t))-{\bar{M}}_{k}^{i}(\lambda_{k}^{i}(t-1))},\;(t=1,2,\cdots )$$

where ${\bar{M}}_{k}^{i}(\lambda_{k}^{i}(t))={\tilde{z}}_{i}^{T}(k){\left(P_{i}^{*}({\hat{z}}_{i}(k/k-1))\right)}^{-1}{\tilde{z}}_{i}(k)$. 

The above iterative process to determine the adaptive fading factor $\lambda_{k}^{i}$ is initialized as $\lambda_{k}^{i}(1)=1$ and $\lambda_{k}^{i}(0)=0$. It will be terminated when the criterion ${\bar{M}}_{k}^{i}[\lambda_{k}^{i}(t)]\leq \chi_{m_{i},\alpha }^{2}$ is satisfied. The calculation process of the Mahalanobis distance-based AFUKF can be summarized as:

**Step** **1:** Initialization
  Initiate the Local filters by presetting the ${\hat{x}}_{i}(0)$ and $P_{i}(0)$ as (2).
**Step** **2:** *Local Filtering*
  (i) Conduct the classical UKF procedures (3)–(10) and calculate the ${\bar{M}}_{k}^{i}[\lambda_{k}^{i}(t)]$.
  (ii) If ${\bar{M}}_{k}^{i}[\lambda_{k}^{i}(t)]\leq \chi_{m_{i},\alpha }^{2}$,
    - Perform the classical UKF procedures (11)–(14) to compute the local state estimations.
    Else,
    - Determine the adaptive fading factor $\lambda_{k}^{i}$ by iteratively computing (22) until the criterion ${\bar{M}}_{k}^{i}[\lambda_{k}^{i}(t)]\leq \chi_{m_{i},\alpha }^{2}$ is satisfied.
    - Then, replace the predicted state covariance matrix $P_{i}(k/k-1)$ of the classical UKF with the modified type $P_{i}^{*}(k/k-1)$ as described by (19).
    - Complete the classical UKF procedures (7)–(14) to update the local state estimations.
  (iii) Repeat Steps (i) and (ii) for the next sample.
**Step** **3:** Complete the local state estimations until all samples are processed.

Each local filter is performed in a parallel manner to generate the local state estimation ${\hat{x}}_{i}(k)$ and the corresponding error covariance $P_{i}(k)$ (i=1,2,⋯,N) by using the Mahalanobis distance-based AFUKF. After this, these local state estimations will be further fused by the UT-based data fusion method, which is to be described in the next section, to obtain the globally optimal state estimation.

### 2.2. Global State Estimation

Suppose that the local state estimation achieved by the *i*th (i=1,2,⋯,N) local filter is ${\hat{x}}_{i}(k)$. According to the linear weighting fusion criterion, the globally optimal state estimation can be described as the following form

(23) $${\hat{x}}^{*}(k)=\sum_{i=1}^{N}\alpha_{i}{\hat{x}}_{i}(k)$$

(24) $$\sum_{i=1}^{N}\alpha_{i}=I_{n}$$

where $\alpha_{i}$ (i=1,2,⋯,N) is the weighting matrix to be determined; and $I_{n}$ represents an *n*-dimensional identity matrix.

Based on the principle of linear minimum variance, the globally optimal state estimation ${\hat{x}}^{*}(k)$ should satisfy the following conditions [14,24]:

**Condition** **1.** ${\hat{x}}^{*}(k)$ *must be the unbiased estimation of* x(k)*, i.e.,* $E\left\{{\hat{x}}^{*}(k)-x(k)\right\}=0$.

**Condition** **2.** ${\hat{x}}^{*}(k)$ *makes* $tr\left\{P^{*}(k)\right\}$ *minimum, where* $P^{*}(k)$ *is the error covariance matrix of* ${\hat{x}}^{*}(k)$*, and* tr{⋅} *is the trace operator.*

**Theorem** **1.** *Denote the local state estimation achieved by the ith (*i=1,2,⋯,N*) local filter by* ${\hat{x}}_{i}(k)$*, and its corresponding error covariance matrix by* $P_{ii}(k)$ *(i.e.,* $P_{i}(k)$*,* i=1,2,…,N *in the local filters). The cross-covariance matrix between the state estimation errors of* ${\hat{x}}_{i}(k)$ *and* ${\hat{x}}_{j}(k)$ *is* $P_{ij}(k)$ *(*i,j=1,2,…,N*;* i≠j*). Define* ${\left(\Sigma \left(k\right)\right)}_{ij}=P_{ij}(k)\in R^{n\times n}$ *(*i,j=1,2,…,N*),* $\bar{E}={\left[I_{n},I_{n},\ldots ,I_{n}\right]}^{T}\in R^{nN\times n}$ *and* $\bar{\alpha }={\left[\alpha_{1},\alpha_{2},\ldots ,\alpha_{N}\right]}^{T}\in R^{nN\times n}$*. Based on the principle of linear minimum variance, the globally optimal state estimation* ${\hat{x}}^{*}(k)$ *is obtained as*

(25) $${\hat{x}}^{*}(k)=\sum_{i=1}^{N}\alpha_{i}{\hat{x}}_{i}(k)$$

*where* $\alpha_{i}$ (i=1,2,⋯,N) *is given by*

(26) $$\bar{\alpha }=\Sigma^{-1}\left(k\right)\bar{E}{\left[{\bar{E}}^{T}\Sigma^{-1}\left(k\right)\bar{E}\right]}^{-1}$$

**Proof.**  As $E\left\{{\hat{x}}_{i}(k)-x(k)\right\}=0$, taking the expectation of (25) and considering the relation $\sum_{i=1}^{N}\alpha_{i}=I_{n}$, we can obtain

(27) $$E\left({\hat{x}}^{*}(k)\right)=\sum_{i=1}^{N}\alpha_{i}E\left({\hat{x}}_{i}(k)\right)=x(k)$$

It can be seen from (27), ${\hat{x}}^{*}(k)$ obtained by (25) is the unbiased estimation of x(k).

Now let us consider Condition 2 to derive the globally optimal state estimation ${\hat{x}}^{*}(k)$. Define the estimation error of the local state estimation ${\hat{x}}_{i}(k)$ (i=1,2,…,N) as

(28) $${\tilde{x}}_{i}(k)=x(k)-{\hat{x}}_{i}(k)$$

Thus, the estimation error of ${\hat{x}}^{*}(k)$ is described as

(29) $${\tilde{x}}^{*}(k)=x(k)-{\hat{x}}^{*}(k)=\sum_{i=1}^{N}\alpha_{i}{\tilde{x}}_{i}(k)$$

According to (29), the error covariance matrix of ${\hat{x}}^{*}(k)$ is

(30) $$P^{*}(k)={\bar{\alpha }}^{T}\Sigma \left(k\right)\bar{\alpha }$$

Applying the Lagrange multiplier to solve the minimum of $tr\left\{P^{*}(k)\right\}$, the Lagrange function is defined as

(31) $$𝕃=tr\{P^{*}\left(k\right)\}+tr\{\Lambda [{\bar{\alpha }}^{T}\bar{E}-I_{n}]\}$$

where $\Lambda \in R^{n\times n}$ is the Lagrange multiplier.

Taking the partial derivative of (31) with respect to $\bar{\alpha }$ and letting it to be zero, we have

(32) $$\Sigma \left(k\right)\bar{\alpha }+\frac{1}{2}\bar{E}\Lambda =0$$

Combining $\sum_{i=1}^{N}\alpha_{i}=I_{n}$ with (32), we readily have

(33) $$\left[\begin{array}{cc} \Sigma \left(k\right) & \bar{E}\\ {\bar{E}}^{T} & 0 \end{array}\right]\left[\begin{array}{c} \bar{\alpha }\\ \frac{1}{2}\Lambda \end{array}\right]=\left[\begin{array}{c} 0\\ I_{n} \end{array}\right]$$

By solving (33), it is verified that

(34) $$\left[\begin{array}{c} \bar{\alpha }\\ \frac{1}{2}\Lambda \end{array}\right]={\left[\begin{array}{cc} \Sigma \left(k\right) & \bar{E}\\ {\bar{E}}^{T} & 0 \end{array}\right]}^{-1}\left[\begin{array}{c} 0\\ I_{n} \end{array}\right]=\left[\begin{array}{c} \Sigma^{-1}\left(k\right)\bar{E}{\left[{\bar{E}}^{T}\Sigma^{-1}\left(k\right)\bar{E}\right]}^{-1}\\ -{\left[{\bar{E}}^{T}\Sigma^{-1}\left(k\right)\bar{E}\right]}^{-1} \end{array}\right]$$

Form (34), we have

(35) $$\bar{\alpha }=\Sigma^{-1}\left(k\right)\bar{E}{\left[{\bar{E}}^{T}\Sigma^{-1}\left(k\right)\bar{E}\right]}^{-1}$$

The proof of Theorem 1 is completed. ☐

It can be seen from (25) and (26), in order to calculate the global optimal state estimation ${\hat{x}}^{*}(k)$, it is necessary to predetermine the matrix Σ(k). In the matrix Σ(k), $P_{ii}(k)$(i=1,2,…,N) can be directly determined by the error covariance matrix of the state estimation in the *i*th local filter. However, it is difficult to calculate the cross-covariance matrix $P_{ij}(k)$ between the state estimation errors of ${\hat{x}}_{i}(k)$ and ${\hat{x}}_{j}(k)$. This paper adopts the UT concept to approximate $P_{ij}(k)$, and the derivation process is shown in Theorem 2.

**Theorem** **2.** *For the multi-sensor system given by (1), the cross-covariance matrix* $P_{ij}(k)$ *between the estimation errors of local state estimations* ${\hat{x}}_{i}(k)$ *and* ${\hat{x}}_{j}(k)$ *(*i,j=1,2,…,N*;* i≠j*) is computed as*

(36) $$\begin{array}{cc} P_{ij}(k) & =\sum_{s=0}^{2n}\omega_{s}(\chi_{s,k/k-1}^{\left(i\right)}-{\hat{x}}_{i}(k/k-1)){\left(\chi_{s,k/k-1}^{\left(j\right)}-{\hat{x}}_{j}(k/k-1)\right)}^{T}\\ & -\left[\sum_{s=0}^{2n}\omega_{s}(\chi_{s,k/k-1}^{\left(i\right)}-{\hat{x}}_{i}(k/k-1)){\left(\gamma_{s,k/k-1}^{\left(j\right)}-{\hat{z}}_{j}(k/k-1)\right)}^{T}\right]K_{j}^{T}(k)\\ & -K_{i}(k)\left[\sum_{s=0}^{2n}\omega_{s}(\gamma_{s,k/k-1}^{\left(i\right)}-{\hat{z}}_{i}(k/k-1)){\left(\chi_{s,k/k-1}^{\left(j\right)}-{\hat{x}}_{j}(k/k-1)\right)}^{T}\right]\\ & +K_{i}(k)\left[\sum_{s=0}^{2n}\omega_{s}(\gamma_{s,k/k-1}^{\left(i\right)}-{\hat{z}}_{i}(k/k-1)){\left(\gamma_{s,k/k-1}^{\left(j\right)}-{\hat{z}}_{j}(k/k-1)\right)}^{T}\right]K_{j}^{T}(k) \end{array}$$

*where $\chi_{s,k/k-1}^{\left(i\right)}$ denotes the sigma point transformed by the nonlinear function f(⋅) in (4) for the *i*th local filter; $\gamma_{s,k/k-1}^{\left(i\right)}$ denotes the sigma point transformed by the nonlinear function $h_{i}(\cdot )$ in (8) for the ith local filter; and s=0,1,⋯,2n represents the order of the transformed sigma points.*

**Proof.**  Define the estimation error of the *i*th local filter as

(37) $$\begin{array}{cc} x(k) & -{\hat{x}}_{i}(k)\\ & =x(k)-\left[{\hat{x}}_{i}(k/k-1)+K_{i}(k)\left(z_{i}(k)-{\hat{z}}_{i}(k/k-1)\right)\right]\\ & =\left(x(k)-{\hat{x}}_{i}(k/k-1)\right)-K_{i}(k)\left(z_{i}(k)-{\hat{z}}_{i}(k/k-1)\right) \end{array}$$

Then, it is verified from (37)

(38) $$\begin{array}{cc} P_{ij}(k) & =E\left\{\left[x(k)-{\hat{x}}_{i}(k)\right]{\left[x(k)-{\hat{x}}_{j}(k)\right]}^{T}\right\}\\ & =E\{\left[\left(x(k)-{\hat{x}}_{i}(k/k-1)\right)-K_{i}(k)\left(z_{i}(k)-{\hat{z}}_{i}(k/k-1)\right)\right]\\ & {\;[\left(x(k)-{\hat{x}}_{j}(k/k-1)\right)-K_{j}(k)\left(z_{j}(k)-{\hat{z}}_{j}(k/k-1)\right)]}^{T}\}\\ & =E\left\{\left[\left(x(k)-{\hat{x}}_{i}(k/k-1)\right)\right]{\left[\left(x(k)-{\hat{x}}_{j}(k/k-1)\right)\right]}^{T}\right\}\\ & -E\left\{\left[\left(x(k)-{\hat{x}}_{i}(k/k-1)\right)\right]{\left[\left(z_{j}(k)-{\hat{z}}_{j}(k/k-1)\right)\right]}^{T}\right\}K_{j}^{T}(k)\\ & -K_{i}(k)E\left\{\left[\left(z_{i}(k)-{\hat{z}}_{i}(k/k-1)\right)\right]{\left[\left(x(k)-{\hat{x}}_{j}(k/k-1)\right)\right]}^{T}\right\}\\ & +K_{i}(k)E\left\{\left[\left(z_{i}(k)-{\hat{z}}_{i}(k/k-1)\right)\right]{\left[\left(z_{j}(k)-{\hat{z}}_{j}(k/k-1)\right)\right]}^{T}\right\}K_{j}^{T}(k) \end{array}$$

Based on the tranformated sigma points $\chi_{s,k/k-1}^{\left(i\right)}$ and $\gamma_{s,k/k-1}^{\left(i\right)}$ (i=1,2,⋯,N), $P_{ij}(k)$ can be approximated by using UT

(39) $$\begin{array}{cc} P_{ij}(k)= & \sum_{s=0}^{2n}\omega_{s}(\chi_{s,k/k-1}^{\left(i\right)}-{\hat{x}}_{i}(k/k-1)){\left(\chi_{s,k/k-1}^{\left(j\right)}-{\hat{x}}_{j}(k/k-1)\right)}^{T}\\ & -\left[\sum_{s=0}^{2n}\omega_{s}(\chi_{s,k/k-1}^{\left(i\right)}-{\hat{x}}_{i}(k/k-1)){\left(\gamma_{s,k/k-1}^{\left(j\right)}-{\hat{z}}_{j}(k/k-1)\right)}^{T}\right]K_{j}^{T}(k)\\ & -K_{i}(k)\left[\sum_{s=0}^{2n}\omega_{s}(\gamma_{s,k/k-1}^{\left(i\right)}-{\hat{z}}_{i}(k/k-1)){\left(\chi_{s,k/k-1}^{\left(j\right)}-{\hat{x}}_{j}(k/k-1)\right)}^{T}\right]\\ & +K_{i}(k)\left[\sum_{s=0}^{2n}\omega_{s}(\gamma_{s,k/k-1}^{\left(i\right)}-{\hat{z}}_{i}(k/k-1)){\left(\gamma_{s,k/k-1}^{\left(j\right)}-{\hat{z}}_{j}(k/k-1)\right)}^{T}\right]K_{j}^{T}(k) \end{array}$$

The proof of Theorem 2 is completed. ☐

## 3. Performance Evaluation and Discussion

A prototype system of INS/GNSS/CNS integration was implemented using the proposed AFUKF-MODF. Simulations and experiments as well as comparison analysis with FKF, UKF-FKF [13], and UKF-MODF [14] were conducted to comprehensively evaluate the performance of the proposed AFUKF-MODF.

### 3.1. System Model of INS/GNSS/CNS Integration

Fundamentally, INS/GNSS/CNS integration generates navigation solutions by utilizing the high-precision GNSS position and velocity to correct the INS velocity and position errors and utilizing the high-precision CNS attitude to correct the INS attitude error.

#### 3.1.1. Process Model

The process model of the INS/GNSS/CNS integrated navigation system is established by combining the INS error equations with the inertial measurement unit (IMU) error equations. 

The navigation frame (*n*-frame) is selected as the E-N-U (East-North-Up) geography frame (*g*-frame). Denote the inertial frame by *i*, the earth frame *e*, the body frame *b* and the INS simulated actual platform frame $n^{\prime }$. The system state vector is defined as

(40) $$x(t)={\left[\phi_{E},\phi_{N},\phi_{U},\delta v_{E},\delta v_{N},\delta v_{U},\delta L,\delta \lambda ,\delta h,\varepsilon_{x}^{b},\varepsilon_{y}^{b},\varepsilon_{z}^{b},\nabla_{x}^{b},\nabla_{y}^{b},\nabla_{z}^{b}\right]}^{T}$$

where $\left(\phi_{E},\phi_{N},\phi_{U}\right)$ is the attitude error, $\left(\delta v_{E},\delta v_{N},\delta v_{U}\right)$ the velocity error, (δL,δλ,δh) the position error, $\left(\varepsilon_{x}^{b},\varepsilon_{y}^{b},\varepsilon_{z}^{b}\right)$ the gyro constant drift, and $\left(\nabla_{x}^{b},\nabla_{y}^{b},\nabla_{z}^{b}\right)$ the accelerometer zero-bias.

The nonlinear attitude and velocity error equations are given as [34,35]

(41) $$\left\{ \begin{array}{c} \dot{\phi }=C_{\omega }^{-1}[(I-C_{n}^{n^{\prime }}){\hat{\omega }}_{in}^{n}+C_{n}^{n^{\prime }}\delta \omega_{in}^{n}-C_{b}^{n^{\prime }}\delta \omega_{ib}^{b}]\\ \delta {\dot{v}}^{n}=\delta g^{n}+[I-{\left(C_{n}^{n^{\prime }}\right)}^{T}]C_{b}^{n^{\prime }}{\hat{f}}^{b}+C_{b}^{n}\delta f^{b}-\\ (2{\hat{\omega }}_{ie}^{n}+{\hat{\omega }}_{en}^{n})\times \delta v^{n}-(2\delta \omega_{ie}^{n}+\delta \omega_{en}^{n})\times v^{n} \end{array}\right.$$

where $\phi ={\left(\phi_{E},\phi_{N},\phi_{U}\right)}^{T}$ and $\delta v^{n}={\left(\delta v_{E},\delta v_{N},\delta v_{U}\right)}^{T}$; $v^{n}={\left(v_{E},v_{N},v_{U}\right)}^{T}$ is the velocity of the vehicle in *n*-frame; $C_{n}^{n^{\prime }}$, $C_{b}^{n^{\prime }}$ and $C_{b}^{n}$ are the rotation matrices; $\delta g^{n}$ is the gravity error; ${\hat{f}}^{b}$ is the measured specific force in the *b*-frame, and it is composed of accelerometer zero-bias $\nabla^{b}$ and its white noise $\omega_{a}^{b}$; $\delta f^{b}$ is the corresponding error; $\delta \omega_{ib}^{b}$ is the measurement error of the gyro, which is composed of gyro constant drift $\varepsilon^{b}$ and its white noise $\omega_{g}^{b}$; $\omega_{ie}^{n}$ is the rotational angular velocity of the earth; $\omega_{en}^{n}$ is the angular velocity of the vehicle relative to the earth; $\omega_{in}^{n}=\omega_{ie}^{n}+\omega_{en}^{n}$ is the relative rotational angular velocity between the *n*-frame and *i*-frame; ${\hat{\omega }}_{ie}^{n}$, ${\hat{\omega }}_{en}^{n}$ and ${\hat{\omega }}_{in}^{n}$ are the actual values of $\omega_{ie}^{n}$, $\omega_{en}^{n}$ and $\omega_{in}^{n}$ in the $n^{\prime }$-frame; and $\delta \omega_{ie}^{n}$, $\delta \omega_{en}^{n}$ and $\delta \omega_{in}^{n}$ represent the corresponding errors. The above parameters can be calculated as

(42) $$\left\{ \begin{array}{l} \omega_{ie}^{n}={\left[\begin{array}{ccc} 0 & \omega_{ie}\cos L & \omega_{ie}\sin L \end{array}\right]}^{T}\\ \delta \omega_{ie}^{n}={\left[\begin{array}{ccc} 0 & -\delta L\omega_{ie}\sin L & \delta L\omega_{ie}\cos L \end{array}\right]}^{T}\\ \omega_{en}^{n}={\left[\begin{array}{ccc} -\frac{v_{N}}{R_{M}+h} & \frac{v_{E}}{R_{N}+h} & \frac{v_{E}\tan L}{R_{N}+h} \end{array}\right]}^{T}\\ \delta \omega_{en}^{n}=\left[\begin{array}{c} -\frac{\delta v_{N}}{R_{M}+h}+\delta h\frac{v_{N}}{{\left(R_{M}+h\right)}^{2}}\\ \frac{\delta v_{E}}{R_{N}+h}-\delta h\frac{v_{E}}{{\left(R_{N}+h\right)}^{2}}\\ \frac{\delta v_{E}\tan L}{R_{N}+h}+\frac{\delta Lv_{x}\sec L^{2}}{R_{N}+h}-\delta h\frac{v_{E}\sec L}{{\left(R_{N}+h\right)}^{2}} \end{array}\right] \end{array}\right.$$

where L and h represent the latitude and altitude of the vehicle; and $R_{M}$ and $R_{N}$ are the median radius and normal radius.

$C_{\omega }^{-1}$ is computed as

(43) $$C_{\omega }^{-1}=\frac{1}{\cos\phi_{E}}\left[\begin{array}{ccc} \cos\phi_{N}\cos\phi_{E} & 0 & \sin\phi_{N}\cos\phi_{E}\\ \sin\phi_{N}\sin\phi_{E} & \cos\phi_{E} & -\cos\phi_{N}\sin\phi_{E}\\ -\sin\phi_{N} & 0 & \cos\phi_{N} \end{array}\right]$$

The position error equation of INS is described by [36]

(44) $$\{\begin{array}{l} \delta \dot{L}=\frac{\delta v_{N}}{R_{M}+h}-\delta h\frac{v_{N}}{{\left(R_{M}+h\right)}^{2}}\\ \delta \dot{\lambda }=\frac{\delta v_{E}\sec L}{R_{N}+h}+\delta L\frac{v_{E}\tan L\sec L}{R_{N}+h}-\delta h\frac{v_{E}\sec L}{{\left(R_{N}+h\right)}^{2}}\\ \delta \dot{h}=\delta v_{U} \end{array}$$

The gyro constant drift $\varepsilon^{b}$ and accelerometer zero-bias $\nabla^{b}$ are commonly described as random constants [14,30], i.e.,

(45) $${\dot{\varepsilon }}_{i}^{b}=0\;(i=x,y,z)$$

(46) $${\dot{\nabla }}_{i}^{b}=0\;(i=x,y,z)$$

According to the selected system state vector, the process model of the INS/GNSS/CNS integration can be established by combining (41)–(46)

(47) $$\dot{x}(t)=\bar{f}\left(x(t)\right)+w(t)$$

where $\bar{f}\left(\cdot \right)$ is a nonlinear function describing the system state equation in continuous form; and $w(t)={\left[{\left(-C_{\omega }^{-1}C_{b}^{n^{\prime }}\omega_{a}^{b}\right)}^{T},{\left(C_{b}^{n}\omega_{g}^{b}\right)}^{T},0_{1\times 9}\right]}^{T}$ is the process noise vector.

By discretizing (47) with the improved Euler formulation [37], the discrete-time process model of the INS/GNSS/CNS integration is obtained as

(48) x(k)=f(x(k−1))+w(k)

where f(⋅) is a nonlinear function describing the system state equation in discrete form; and w(k) is the discrete-time process noise vector.

#### 3.1.2. Measurement Model of INS/GNSS Subsystem

Take the difference between INS and GNSS in terms of velocity and position as the measurement of the INS/GNSS subsystem, i.e.,

(49) $$z_{1}(k)={\left[\begin{array}{llllll} v_{EI}-v_{EG} & v_{NI}-v_{NG} & v_{UI}-v_{UG} & L_{I}-L_{G} & \lambda_{I}-\lambda_{G} & h_{I}-h_{G} \end{array}\right]}^{T}$$

where ${\left(v_{EI},v_{NI},v_{UI}\right)}^{T}$ and ${\left(L_{I},\lambda_{I},h_{I}\right)}^{T}$ are the velocity and position output by INS; and ${\left(v_{EG},v_{NG},v_{UG}\right)}^{T}$ and ${\left(L_{G},\lambda_{G},h_{G}\right)}^{T}$ are the velocity and position achieved by GNSS. 

Then, the measurement model of the INS/GNSS subsystem can be described as [11,24]

(50) $$\begin{array}{cc} z_{1}(k) & =H_{1}(k)x(k)+v_{1}(k)\\ & \;=\left[\begin{array}{c} H_{v}(k)\\ H_{P}(k) \end{array}\right]x(k)+\left[\begin{array}{c} v_{v}(k)\\ v_{P}(k) \end{array}\right] \end{array}$$

where $H_{v}(k)=[0_{3\times 3},I_{3\times 3},0_{3\times 9}]$, $H_{P}(k)=[0_{3\times 6},diag(R_{M},R_{N}\cos L,1),0_{3\times 6}]$; and $v_{v}(k)$ and $v_{P}(k)$ are the measurement noises, which correspond to the velocity and position errors of GNSS, respectively.

#### 3.1.3. Measurement Model of INS/CNS Subsystem

CNS provides high-precise attitude information for a vehicle through the star sensor. Take the difference between INS and CNS in terms of attitude as the measurement of the INS/CNS subsystem, i.e.,

(51) $$z_{2}(k)=\left[\begin{array}{ccc} \phi_{EI}-\phi_{EC} & \phi_{NI}-\phi_{NC} & \phi_{UI}-\phi_{UC} \end{array}\right]$$

where ${\left(\phi_{EI},\phi_{NI},\phi_{UI}\right)}^{T}$ and ${\left(\phi_{EC},\phi_{NC},\phi_{UC}\right)}^{T}$ are the attitude information obtained by INS and CNS, respectively.

Accordingly, the measurement model of the INS/CNS subsystem is constructed as [11,24]

(52) $$z_{2}(k)=H_{2}(k)x(k)+v_{2}(k)$$

where $H_{2}(k)=[I_{3\times 3},0_{3\times 12}]$, and $v_{2}(k)$ is the measurement noise corresponding to the measurement error of the star sensor.

The INS/GNSS/CNS integrated navigation system is a multi-sensor nonlinear system with 2 local filters (*N* = 2). The nonlinear system model, which is generally described by (1), is now specified by (48), (50) and (52) for the specific INS/GNSS/CNS integrated navigation system. The system models of the INS/GNSS and INS/CNS subsystems are described by (48) and (50), and (48) and (52), respectively. Based on these, the AFUKF based on Mahalanobis distance described in Section 2.1.2 serves as the local filters to calculate the local state estimations of the INS/GNSS and INS/CNS subsystems. Subsequently, both local state estimations of the INS/GNSS and INS/CNS subsystems are fused by the UT-based multi-sensor optimal data fusion described in Section 2.2 to calculate the globally optimal state estimation for the INS/GNSS/CNS integration.

### 3.2. Simulations and Analysis

**Figure 2:**
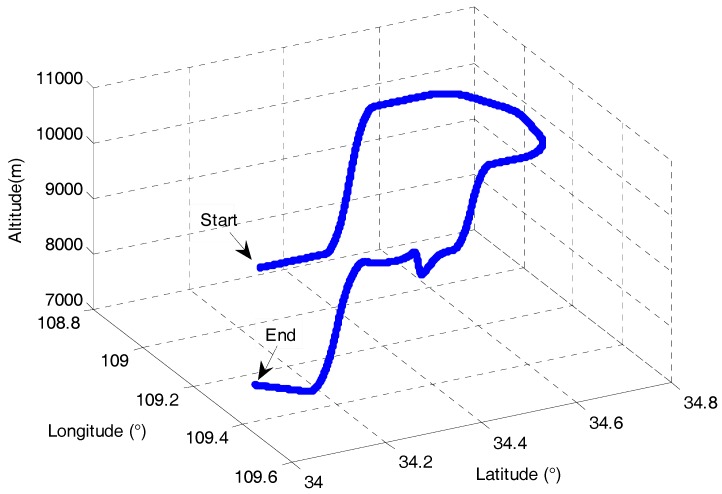
Flight trajectory of the UAV.

Monte Carlo simulations were conducted to comprehensively evaluate the performance of the proposed AFUKF-MODF for the dynamic flight of an unmanned aerial vehicle (UAV) using the INS/GNSS/CNS integration for navigation and positioning. The flight trajectory, which involves various maneuvers such as climbing, pitching, rolling and turning, is shown in Figure 2. The simulation parameters are listed in Table 1. Both filtering period and fusion period were 1 s. The Monte Carlo simulations were carried out 100 times.

The initial state error covariances and process noise covariance for the local filters are set as

(53) $$P_{i}(0)=diag\left[{\left(1^{\prime }\right)}^{2},{\left(1^{\prime }\right)}^{2},{\left(1.5^{\prime }\right)}^{2},{\left(0.4\;m/s\right)}^{2}I_{3\times 3},{\left(10\;m\right)}^{2},{\left(10\;m\right)}^{2},{\left(15\;m\right)}^{2},{\left(0.1^{\circ }/h\right)}^{2}I_{3\times 3},{\left(1\times 10^{-3}\;g\right)}^{2}I_{3\times 3}\right]\;(i=1,2)$$

(54) $$Q=diag\left[{\left(0.05^{\circ }/\sqrt{h}\right)}^{2}I_{3\times 3},{\left(1\times 10^{-4}\;g\cdot \sqrt{s}\right)}^{2}I_{3\times 3},0_{9\times 9}\right]$$

However, $P_{i}(0)$ (i=1,2) and Q were enlarged to the twice of their initial values for both FKF and UKF-FKF to eliminate the correlation between the two local state estimations by the use of covariance upper bound. Moreover, the process model of the INS/GNSS/CNS integration should be linearized in local fitering processes for the FKF. 

The measurement noise covariances are set as

(55) $$R_{1}=diag\left[{\left(0.05\;m/s\right)}^{2}I_{3\times 3},{\left(5\;m\right)}^{2},{\left(5\;m\right)}^{2},{\left(8\;m\right)}^{2}\right]$$

(56) $$R_{2}={\left(5^{\prime \prime }\right)}^{2}I_{3\times 3}$$

In order to evaluate the performance of the proposed AFUKF-MODF in terms of process-modeling error, the following process-modeling error is introduced to the process model of the INS/GNSS/CNS integration during the time interval from 400 s to 600 s

(57) $$\Delta x={\left[0_{1\times 3},0.02\;m/s,0.02\;m/s,0.02\;m/s,(2\times 10^{-6}{)}^{\prime },(2\times 10^{-6}{)}^{\prime },5\;m,0_{1\times 6}\right]}^{T}$$

Accordingly, the process model used in the local filters is

(58) $$\left\{ \begin{array}{cc} x(k)=f\left(x(k-1)\right)+w(k) & ,\;\mathrm{other}\;\mathrm{time}\;\mathrm{intervals}\\ x(k)=f(x(k-1))+\Delta x+w(k) & ,(400\;s,600\;s) \end{array}\right.$$

To identify the above process-modeling error, $\chi_{m_{1},\alpha }^{2}$ and $\chi_{m_{2},\alpha }^{2}$ used in the proposed AFUKF-MODF for the INS/GNSS subsystem and INS/CNS subsystem were chosen as 12.592 and 7.815, respectively. These values are resulted from the $\chi^{2}$ distribution when the reliability level is 95% (α=0.05) and the degrees of freedom are 6 and 3, respectively.

For comparison analysis, simulation trials were conducted at the same conditions by using FKF, UKF-FKF, UKF-MODF and AFUKF-MODF, respectively. Moreover, the overall estimation error is adopted to evaluate the navigation accuracy in the simulation analysis for the four fusion methods. It is defined as the norm of the navigation parameters estimation error [38]

(59) $$\left\|\Delta x\right\|=\sqrt{\Delta x_{E}^{2}+\Delta x_{N}^{2}+\Delta x_{U}^{2}}$$

where $\Delta x_{E}$, $\Delta x_{N}$ and $\Delta x_{U}$ are the components of Δx in East, North and Up, respectively.

**Figure 3:**
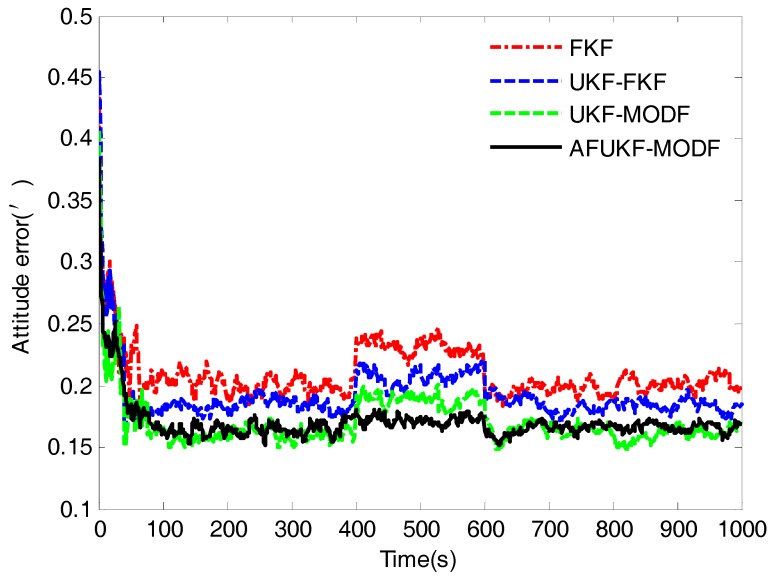
The RMSEs of overall attitude errors obtained by FKF, UKF-FKF, UKF-MODF and AFUKF-MODF for the simulation case.

**Figure 4:**
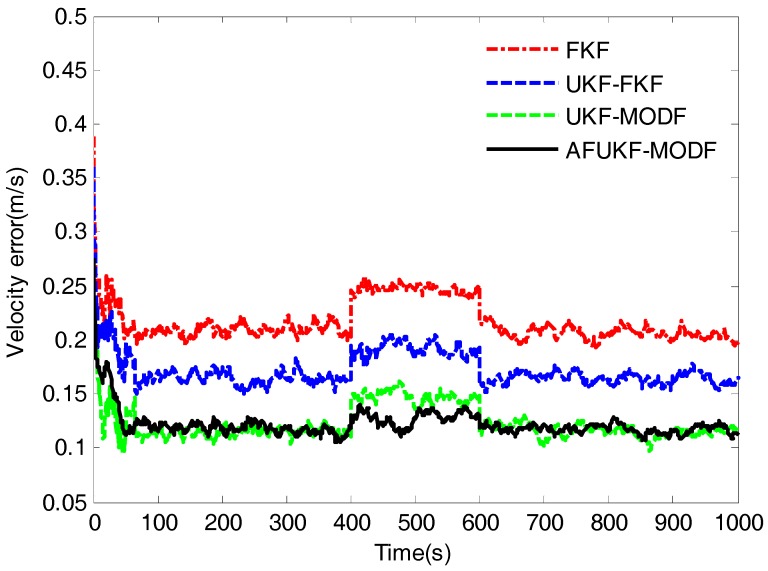
The RMSEs of overall velocity errors obtained by FKF, UKF-FKF, UKF-MODF and AFUKF-MODF for the simulation case.

**Figure 5:**
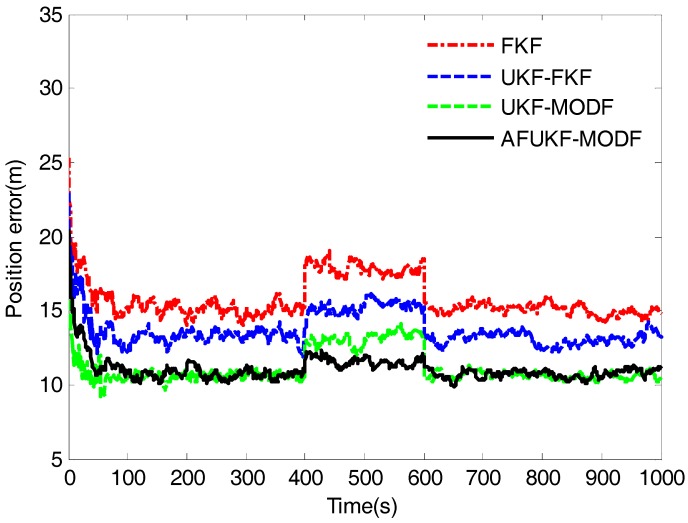
The RMSEs of overall position errors obtained by FKF, UKF-FKF, UKF-MODF and AFUKF-MODF for the simulation case.

Figure 3, Figure 4 and Figure 5 depict the root mean squared errors (RMSEs) of overall attitude errors, velocity errors and position errors by the above four fusion methods, respectively. During the time intervals (0 s, 400 s) and (600 s, 1000 s) without process-modeling error involved, FKF has poor navigation accuracy compared to UKF-FKF, UKF-MODF and AFUKF-MODF. Its estimation RMSEs in attitude, velocity and position are around $0.2010^{\prime }$, 0.2084 m/s and 15.1129 m. This is because the use of covariance upper bound and the linearization of the system model cause suboptimal fusion performance. UKF-FKF adopts UKF to calculate the local state estimations, thus effectively refraining from the error caused by the linearization of system model and leading to the improved fusion accuracy. Its estimation RMSEs in attitude, velocity and position are around $0.1844^{\prime }$, 0.1647 m/s and 13.1980 m. However, due to the use of covariance upper bound, the fusion results of UKF-FKF are still suboptimal. Different to FKF and UKF-FKF, both UKF-MODF and AFUKF-MODF are directly derived based on the principle of linear minimum variance, avoiding the use of covariance upper bound in the local filters. Thus, the fusion results obtained by both UKF-MODF and AFUKF-MODF are globally optimal, which are much more accurate than those by FKF and UKF-FKF. The estimation RMSEs in attitude, velocity and position are around $0.1612^{\prime }$, 0.1155 m/s and 10.6099 m for UKF-MODF, and $0.1643^{\prime }$, 0.1176 m/s and 10.7212 m for AFUKF-MODF. Moreover, since there is no process-modeling error identified, the proposed AFUKF-MODF does not incorporate the adaptive fading factor into the local estimation processes. Accordingly, its navigation accuracy is close to that of UKF-MODF during these time intervals.

During the time interval (400 s, 600 s), due to the influence of the introduced process-modeling error, the navigation accuracy of FKF, UKF-FKF and UKF-MODF degrades seriously. This is because these three methods do not have the ability to resist the influence of process-modeling error on the fusion solution. The estimation RMSEs in attitude, velocity and position are around $0.2319^{\prime }$, 0.2471 m/s and 17.8467 m for FKF; $0.2078^{\prime }$, 0.1917 m/s and 15.2703 m for UKF-FKF; and $0.1887^{\prime }$, 0.1472 m/s and 13.1225 m for UKF-MODF. In contrast, the proposed AFUKF-MODF has higher navigation accuracy than FKF, UKF-FKF and UKF-MODF during this time interval. Its estimation RMSEs in attitude, velocity and position are around $0.1702^{\prime }$, 0.1280 m/s and 11.5798 m. This is because the proposed AFUKF-MODF can identify the process-modeling error according to the hypothesis testing theory and effectively refrain from the influence of process-modeling error on the fusion solution by adjusting the time-varying adaptive fading factor incorporated in the predicted state covariance matrix, leading to a strong adaptability and robustness.

The mean RMSEs of the over attitude errors, velocity errors and position errors obtained by FKF, UKF-FKF, UKF-MODF and AFUKF-MODF for the time interval (400 s, 600 s) and the other time intervals are listed in Table 2. The results in Table 2 also verify that the proposed AFUKF-MODF has a better adaptability and robustness than the other methods, thus leading to improved navigation accuracy for the INS/GNSS/CNS integration.

The above simulations and analysis demonstrate that the proposed AFUKF-MODF can overcome the limitation of using covariance upper bound and provide globally optimal fusion results. It can also enhance the adaptability and robustness against process-modeling error to overcome the limitation of UKF-MODF, thus leading to higher navigation accuracy than FKF, UKF-FKF and UKF-MODF in the presence of process-modeling error.

### 3.3. Experiments and Analysis

**Figure 6:**
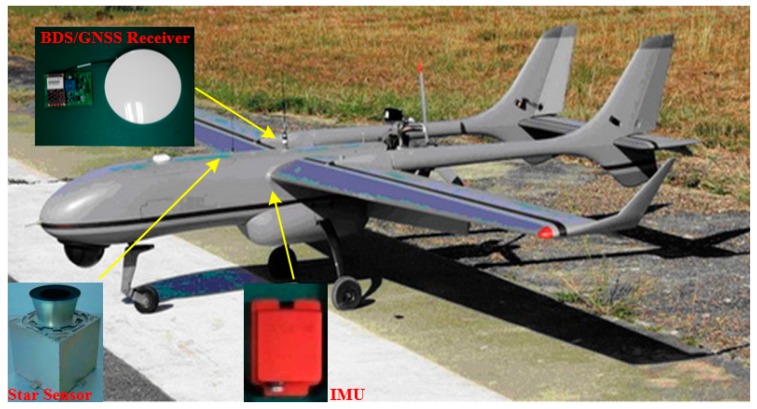
Navigation setup of the UAV.

Practical experiments were also conducted to evaluate the performance of the proposed AFUKF-MODF by observing the flight of an UAV. As shown in Figure 6, the UAV uses an INS/GNSS/CNS integration system for navigation. This navigation system includes a MTi-100 IMU, a Hemisphere P307 BDS/GNSS receiver and a SODERN SED26 star sensor. The main parameters of the above three devices are listed in Table 3, Table 4 and Table 5. Moreover, another Hemisphere P370 BDS/GNSS receiver, which was placed at a local reference station (around 1km from the initial UAV position), was used along with the one mounted on the UAV to provide the differential GPS (DGPS) data. The maximal distance between the UAV and local reference station was less than 60 km to achieve the position accuracy of less than 0.1 m from the DGPS via post difference processing. The DGPS data were used as the reference values to evaluate the positioning error of the INS/GNSS/CNS integrated system.

The UAV flight test was conducted at the City of Yanliang in Shaanxi, China. The experimental navigation data were selected from the UAV flight test within a continuous time period of 1000 s, where different maneuvers were involved. Within the selected UAV flight test, the UAV initial position was at East longitude 109.217°, North latitude 34.647° and altitude 3525 m. The UAV initial velocity was 180 m/s, 150 m/s and 50 m/s in East, North and Up, respectively. The other initial parameters were set identically to those in the simulation case. The filtering periods of the local filters, i.e., the INS/GNSS and INS/CNS subsystems, were 1 s. The period for fusion of local state estimations was also 1 s.

**Figure 7:**
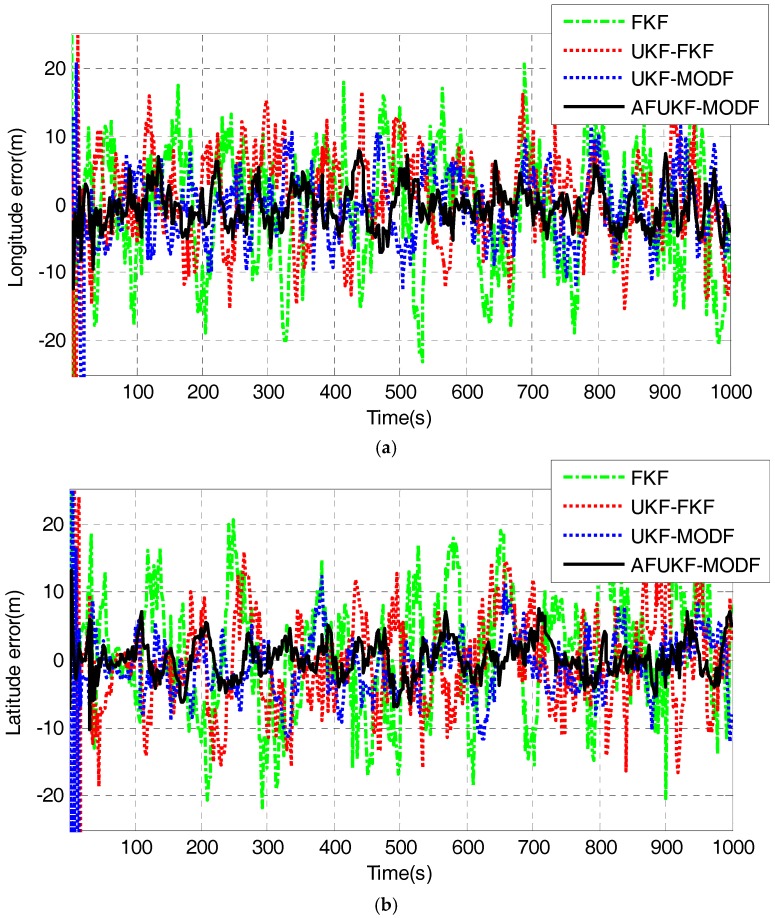

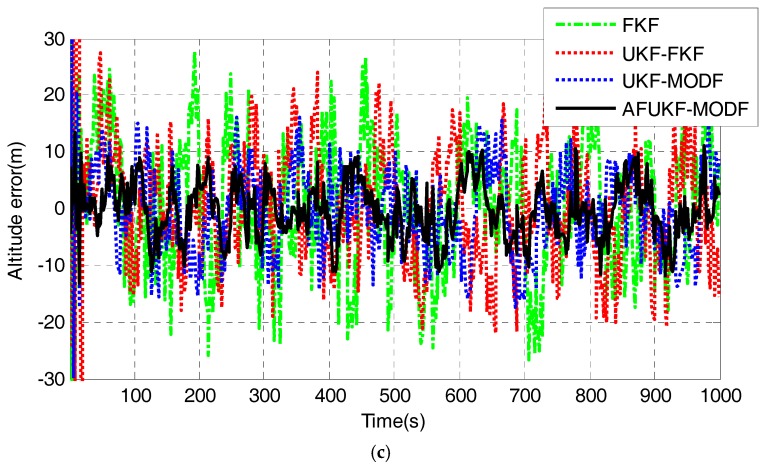
The position errors obtained by FKF, UKF-FKF, UKF-MODF and AFUKF-MODF for the UAV experiment test: (**a**) Longitude error; (**b**) Latitude error; (**c**) Altitude error.

Due to the complexity of the dynamic flight environment, the process model of the INS/GNSS/CNS integration involves modeling error during the UAV flight process. Figure 7 illustrates the UAV position errors obtained by FKF, UKF-FKF, UKF-MODF and AFUKF-MODF, respectively. As shown in Figure 7, the position error achieved by FKF is relatively large due to the influence of process-modeling error, the use of the covariance upper bound and the linearization of the process model. During the time period (100 s, 1000 s), the position errors in longitude, latitude and altitude for FKF are within (−23.0174 m, 20.7557 m), (−21.7712 m, 20.6464 m) and (−26.7975 m, 27.7306 m), respectively. UKF-FKF improves the fusion accuracy of FKF by adopting UKF to calculate the local state estimations, leading to the longitude, latitude and altitude errors within (−15.1817 m, 16.5516 m), (−16.5740 m, 15.9417 m) and (−21.8423 m, 24.0462 m). UKF-MODF can provide globally optimal fusion results based on the principle of linear minimum variance. Thus, it has higher navigation accuracy than FKF and UKF-FKF, leading to the longitude, latitude and altitude errors within (−12.0942 m, 11.8042 m), (−12.0311 m, 12.2480 m) and (−16.9458 m, 15.9384 m). However, its position error curve still has pronounced oscillations due to the influence of the process-modeling error. In contrast, the position errors in longitude, latitude and altitude for the proposed AFUKF-MODF are within (−7.1247 m, 7.8715 m), (−7.0576 m, 7.5495 m) and (−11.8677 m, 11.2610 m), which are much smaller than those by FKF, UKF-FKF and UKF-MODF. This is because the proposed AFUKF-MODF has the capability to inhibit the disturbances of process-modeling error.

Table 6 lists the mean absolute errors (MAEs) and standard deviations (STDs) of the position errors achieved by FKF, UKF-FKF, UKF-MODF and AFUKF-MODF. It can be seen that the MAE and STD of the position errors by the proposed AFUKF-MODF are also much smaller than those of the other three methods.

The above experimental results and analysis demonstrate that the proposed AFUKF-MODF enhances the adaptability and robustness of multi-sensor data fusion in presence of process-modeling errors, leading to improved navigation accuracy for INS/GNSS/CNS integration in comparison with FKF, UKF-FKF and UKF-MODF.

## 4. Conclusions

This paper presents a novel AFUKF-MODF to improve the fusion adaptability and robustness for multi-sensor nonlinear stochastic systems against process-modeling error. The contributions of this paper are: (i) an AFUKF based on the Mahalanobis distance is developed to improve the adaptability and robustness of local state estimations against process-modeling error; and (ii) an UT-based multi-sensor optimal data fusion for the case of *N* local filters is also established to achieve the globally optimal state estimation via fusion of local state estimations. Simulations and practical experiments as well as comparison analysis demonstrate that the proposed AFUKF-MODF not only resists the disturbance of process-modeling error on the fusion solution, leading to improved adaptability and robustness for multi-sensor data fusion, but it also provides globally optimal data fusion results in the sense of linear minimum variance. The achieved fusion accuracy is much higher than that of FKF, UKF-FKF and UKF-MODF for INS/GNSS/CNS integration.

Future research work will focus on two aspects. One is the improvement of the proposed AFUKF-MODF. It is expected to combine the proposed AFUKF-MODF with artificial intelligence technologies such as machine learning, pattern recognition and neural networking to intelligently identify and compensate for system modeling error. The other is on the applications of the proposed AFUKF-MODF to address the multi-sensor data fusion problems in other fields such as target tracking, fault diagnosis and signal processing.

## Figures and Tables

**Table 1:** Simulation parameters.

Parameters			Values
Initial parameters	Initial position (longitude-latitude-altitude)		(108.997°, 34.246°, 5000 m)
	Initial velocity (east-north-up)		(0 m/s, 150 m/s, 0 m/s)
	Initial attitude (pitch-roll-yaw)		(0°, 0°, 0°)
Initial parameter errors	Initial position error (longitude-latitude-altitude)		(10 m, 10 m, 15 m)
	Initial velocity error (east-north-up)		(0.4 m/s, 0.4 m/s, 0.4 m/s)
	Initial attitude error (pitch-roll-yaw)		(1′, 1′, 1.5′)
INS parameters	Gyro parameters	Constant drift	0.1 °/h
		Random walk coefficient	0.05 °/√h
		Sampling frequency	20 Hz
	Accelerometer parameters	Zero-bias	1 × 10^−3^ g
		Random walk coefficient	1 × 10^−4^ g×√s
		Sampling frequency	20 Hz
GNSS parameters	Horizontal position error (RMS)		5 m
	Altitude error (RMS)		8 m
	Velocity error (RMS)		0.05 m/s
	Data update rate		1 Hz
CNS parameters	Attitude error (RMS)		5″
	Data update rate		1 Hz
Simulation time	1000 s		

**Table 2:** Mean RMSEs of the overall estimation errors obtained by FKF, UKF-FKF, UKF-MODF and AFUKF-MODF for the simulation case.

Data fusion Methods	Navigation Errors	Mean RMSE	
		(400 s, 600 s)	The Other Time Intervals
FKF	Attitude error (′)	0.2319	0.2010
	Velocity error (m/s)	0.2471	0.2084
	Position error (m)	17.8467	15.1129
UKF-FKF	Attitude error (′)	0.2078	0.1844
	Velocity error (m/s)	0.1917	0.1647
	Position error (m)	15.2703	13.1980
UKF-MODF	Attitude error (′)	0.1887	0.1612
	Velocity error (m/s)	0.1472	0.1155
	Position error (m)	13.1225	10.6099
AFUKF-MODF	Attitude error (′)	0.1702	0.1643
	Velocity error (m/s)	0.1280	0.1176
	Position error (m)	11.5798	10.7212

**Table 3:** Noise parameters of MTi-100 IMU.

Noise Parameters		Values
Gyro	Constant drift	10°/h
	Random walk coefficient	$0.6^{\circ }/\sqrt{h}$
Accelerometer	Zero-bias	40 μg
	Random walk coefficient	$80\;\mu g\times \sqrt{s}$

**Table 4:** Main parameters of Hemisphere P307 BDS/GNSS receiver.

Feature Parameters	Values
Satellite signals	BDS(B1, B2, B3), GPS(L1, L2), GLONASS(G1, G2)
Horizontal position error (RMS)	1.2 m
Altitude error (RMS)	3 m
Velocity error (RMS)	0.02 m/s
Data update rate	20 Hz

**Table 5:** Main parameters of SODERN SED26 star sensor.

Feature Parameters	Values
Field of view (FOV)	15.437° × 15.437° (Wide FOV)
Observable star number	≥6
Attitude error (RMS)	$5^{\prime \prime }$
Data update rate	10 Hz

**Table 6:** MAEs and STDs of the position errors obtained by FKF, UKF-FKF, UKF-MODF and AFUKF-MODF for the UAV experiment test.

Data Fusion Methods		Position		
		Longitude	Latitude	Altitude
FKF	MAE (m)	7.2719	7.3707	9.0803
	STD (m)	8.8325	8.9150	11.1273
UKF-FKF	MAE (m)	5.4530	5.5924	8.0972
	STD (m)	6.5879	6.8447	9.8784
UKF-MODF	MAE (m)	3.8872	3.8996	6.1051
	STD (m)	4.8051	4.8142	7.3485
AFUKF-MODF	MAE (m)	2.3503	2.3610	4.0656
	STD (m)	2.9015	2.9225	4.9706

## Abbreviations

FKF	Federated Kalman filter
UKF	Unscented Kalman filter
UT	Unscented transformation
UKF-FKF	Unscented Kalman filter-based federated Kalman filter
UKF-MODF	Unscented Kalman filter-based multi-sensor optimal data fusion
AFUKF	Adaptive fading unscented Kalman filter
AFUKF-MODF	Adaptive fading unscented Kalman filter-based multi-sensor optimal data fusion
INS	Inertial navigation system
GNSS	Global navigation Satellite System
CNS	Celestial navigation system
IMU	Inertial measurement unit
E-N-U	East-north-up
UAV	Unmanned aerial vehicle
RMS/RMSE	Root mean squared error
BDS	Beidou system
GPS	Global positioning system
DGPS	Differential global positioning system
FOV	Field of view
MAE	Mean absolute error
STD	Standard deviation
